# Cleaning the Label of Cured Meat; Effect of the Replacement of Nitrates/Nitrites on Nutrients Bioaccessibility, Peptides Formation, and Cellular Toxicity of In Vitro Digested Salami

**DOI:** 10.3390/ijms232012555

**Published:** 2022-10-19

**Authors:** Mattia Di Nunzio, Cecilia Loffi, Serena Montalbano, Elena Chiarello, Luca Dellafiora, Gianfranco Picone, Giorgia Antonelli, Tullia Tedeschi, Annamaria Buschini, Francesco Capozzi, Gianni Galaverna, Alessandra Bordoni

**Affiliations:** 1 Department of Food, Environmental and Nutritional Sciences (DeFENS), University of Milan, Via Celoria 2, 20133 Milan, Italy; 2 Department of Food and Drugs, University of Parma, Parco Area delle Scienze 27/A, 43124 Parma, Italy; 3 SSICA-Stazione Sperimentale per l’Industria delle Conserve Alimentari, Viale Faustino Tanara 31/A, 43121 Parma, Italy; 4 Department of Chemistry, Life Sciences and Environmental Sustainability, University of Parma, Parco Area delle Scienze 11/A, 43124 Parma, Italy; 5 COMT (Interdepartmental Centre for Molecular and Translational Oncology), University of Parma, Parco Area delle Scienze 11/A, 43124 Parma, Italy; 6 Department of Agricultural and Food Sciences (DISTAL), University of Bologna, Piazza Goidanich 60, 47521 Cesena, Italy; 7 Interdepartmental Centre for Industrial Agri-Food Research (CIRI), University of Bologna, Piazza Goidanich 60, 47521 Cesena, Italy

**Keywords:** nitrate, nitrite, processed meat, in vitro digestion, bioaccessibility, bioactive peptides, cellular toxicity

## Abstract

Curing salts composed of mixtures of nitrates and nitrites are preservatives widely used in processed meats. Despite many desirable technological effects, their use in meat products has been linked to methemoglobinemia and the formation of nitrosamines. Therefore, an increasing “anti-nitrite feeling” has grown among meat consumers, who search for clean label products. In this view, the use of natural compounds as alternatives represents a challenge for the meat industry. Processing (including formulation and fermentation) induces chemical or physical changes of food matrix that can modify the bioaccessibility of nutrients and the formation of peptides, impacting on the real nutritional value of food. In this study we investigated the effect of nitrate/nitrite replacement with a combination of polyphenols, ascorbate, and nitrate-reducing microbial starter cultures on the bioaccessibility of fatty acids, the hydrolysis of proteins and the release of bioactive peptides after in vitro digestion. Moreover, digested salami formulations were investigated for their impacts on cell proliferation and genotoxicity in the human intestinal cellular model (HT-29 cell line). The results indicated that a replacement of synthetic nitrates/nitrites with natural additives can represent a promising strategy to develop innovative “clean label” salamis without negatively affecting their nutritional value.

## 1. Introduction

Meat is a high-protein, mineral-rich food that also contains several vitamins, particularly type B ones [1]. Processed meat is described as meat that has been cured, salted, or smoked (e.g., ham or bacon) to increase its shelf-life and/or color, and taste [2]. Between 66% and 99% of Europeans consume processed meat, with an average amount consumed per day ranging between 10 and 80 g [3]. Most processed meats contain nitrites (NO_2_^−^) and nitrates (NO_3_^−^) [4], which are authorized as food additives in the European Union under Commission Regulation (EU) No 1129/2011. The addition of nitrates/nitrites has positive traits, including color fixation, flavor enhancement, antioxidant activity, and antimicrobial (*Clostridium botulinum*) preservation [5]. However, their use in meat products is restricted in many countries. In fact, nitrites and nitrates react with amines produced by the decomposition of proteins [6,7,8], resulting in the production of nitrosamines, carcinogens and inflammatory agents.

Consumers’ interest in food ingredients and production methods is on the rise, and the “clean label” trend has forced the food industry to exclude some synthetic additives and replace them with more “natural” ones [9]. As nitrates/nitrites remain among the food additives most feared by consumers [10], the use of natural compounds as alternatives for their full/partial replacement represents a challenge for the meat industry. Although ideal substitutes of synthetic nitrates/nitrites have not yet been found [11,12], some candidates have been recently evidenced. Their use, alone or in combination, could make it possible to produce healthier processed meat products with good sensory characteristics.

In an effort to produce healthier meat products, it must be considered that processing (including formulation and fermentation) deeply modifies both the content and bioaccessibility of nutrients [13,14]. Bioaccessibility, i.e., the fraction of the total amount of a substance that is released from the food matrix during digestion and potentially becomes available for absorption [15], can be modified by changes in the supramolecular architecture and in the network of interactions between molecules, as well as the location of nutrients within compartments, with an impact on the nutritional value of food [16]. Indeed, in a recent work we reported the impact of different sodium chloride content and ripening time on the kinetics of protein hydrolysis and on the formation of peptides and small organic compounds during in vitro digestion of Parmigiano Reggiano cheese [17].

The aim of the present study was to investigate whether a modification in salami processing that allows the replacement of synthetic nitrates/nitrites affects the bioaccessibility of fatty acid, the hydrolysis of proteins, and the release of bioactive peptides after in vitro digestion of the final products. Two innovative formulations not containing nitrites were prepared: the first (SA) was supplemented with nitrate-reducing microbial starter cultures (MSC) and sodium ascorbate (0.3%); the second (SMA) was added with MSC, sodium ascorbate (0.3%) and plant extracts from grapeseed, green tea and olive. The two innovative formulations were compared with the “positive control” (C-NO_2_) added with sodium nitrite, potassium nitrate and MSC, and the “negative control” (C-0) containing neither MSC nor additives (nitrite, polyphenols and ascorbate). The sensory characteristics (color, texture and rancid flavor) of the innovative formulations were comparable to those of the positive control, without negative sensory properties induced by the presence of the plant extracts.

Conventional and experimental salami formulations were subjected to in vitro static gastrointestinal digestion according to the INFOGEST protocol, and the kinetics of fatty acids and protein release from the food matrix were followed by sampling at the end of the gastric phase, as well as in the middle and at the end of the intestinal phase. Digested samples were also analyzed by high-resolution nuclear magnetic resonance (HR-NMR) and liquid chromatography coupled with high-resolution mass spectrometry (LC–HRMS), and the formation of bioactive peptide sequences was demonstrated using a bioinformatic methodology. To further address health concern on processed meat and nitrite use, the digested samples were analyzed for their impact on cell proliferation and genotoxicity in a human colon cancer cell line (HT-29), currently used to study the relationship between food and cellular physiology/metabolism in vitro [18].

## 2. Results

### 2.1. Fatty Acid Composition and Bioaccessibility

As reported by Herranz et al. [19], the major fatty acids in undigested salami were oleic > palmitic > stearic > linoleic acid, which accounted for approximately 94% of the total fatty acids. No differences were detected between the different formulations with the exception of minor fatty acids (myristic, α-linolenic, and gondoic acid) (Table S1).

The bioaccessibility of fatty acids increased during digestion, particularly during the intestinal phase (Table 1). Although the maximum total bioaccessibility was already achieved after 60 min of duodenal digestion in C0 and SMA, at the end of digestion (D120) it was similar in all formulations. Comparing the different salami formulations at each digestion time, at D60, the bioaccessibility of stearic acid was lower in CNO_2_ than SMA (Table S2).

### 2.2. Protein Hydrolysis

**Figure 1:**
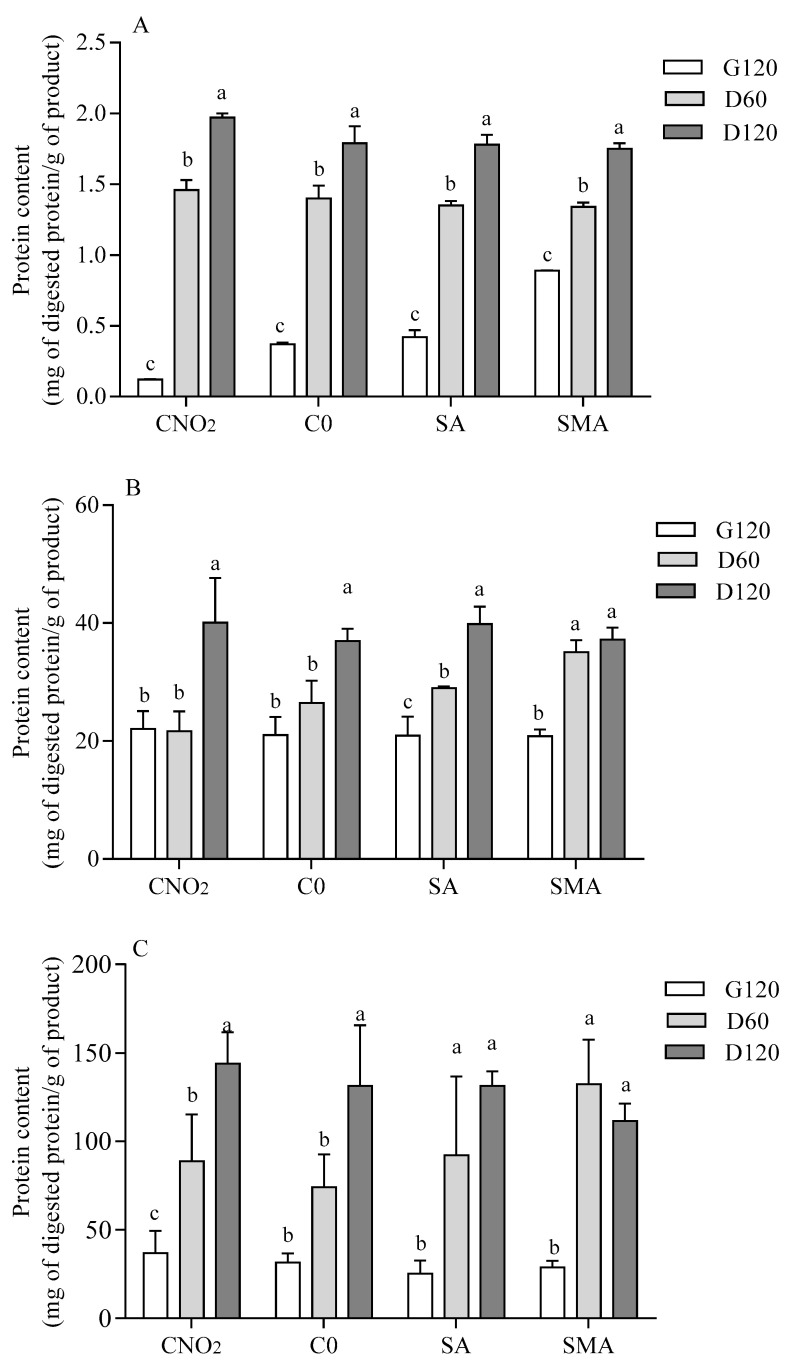
Time-course of protein hydrolysis in the different salami formulations. Protein content was assessed by OPA (**A**), Coomassie assay (**B**), and absorbance at 280 nm (**C**), and it is expressed as milligrams of protein digested/gram of product. Data are means ± SD of in vitro digestion of three independent samples analyzed in triplicate. Protein content is expressed as milligram of protein digested/gram of product. Statistical analysis was by one-way ANOVA (always *p* < 0.05) with Tukey’s post-hoc test comparing each salami formulation at the three digestion time points (different letters indicate statistical significance). G120: end of gastric phase; D60: 60 min of duodenal phase; D120: end of duodenal phase; C-NO_2_: salami with sodium nitrite, potassium nitrate and with nitrate-reducing microbial starter cultures; C-0: salami containing neither nitrate-reducing microbial starter cultures nor additives (nitrite, polyphenols and ascorbate); SA: salami with nitrate-reducing microbial starter cultures and sodium ascorbate; SMA: salami with nitrate-reducing microbial starter cultures, sodium ascorbate and plant extracts.

Protein content in the undigested salami was similar: C-NO_2_ = 32.4 ± 0.9%; C0 = 33.9 ± 1.5%; SA = 31.8 ± 0.7%; SMA = 32.6 ± 0.5% (one-way ANOVA *p* = n.s.; Tukey’s post-hoc test: n.s.). Protein hydrolysis during digestion was evaluated by three different spectrophotometric methods (OPA, Coomassie, and absorbance at 280 nm), all showing a time-dependent release of amino acids/peptides/proteins from the food matrix (Figure 1). Although the three assays have a different ability to detect protein fragments with different molecular masses [20,21], at D120 the same amino acid/peptide/protein concentration was detected in all samples by all methods. Nonetheless, some differences in the time course of protein hydrolysis were found between the formulations. Indeed, SA and SMA already achieved the highest protein hydrolysis at mid-duodenal digestion (D60) as assessed by absorbance at 280 nm and Coomassie assay (for SMA only). Comparing the different salami formulations at each digestion point, no differences were found when measuring absorbance at 280 nm. On the contrary, a different efficiency of protein hydrolysis was highlighted by the OPA assay at G120 and by the Coomassie assay at D60 (Figure S1).

### 2.3. HR ^1^H-NMR Spectroscopy

**Figure 2:**
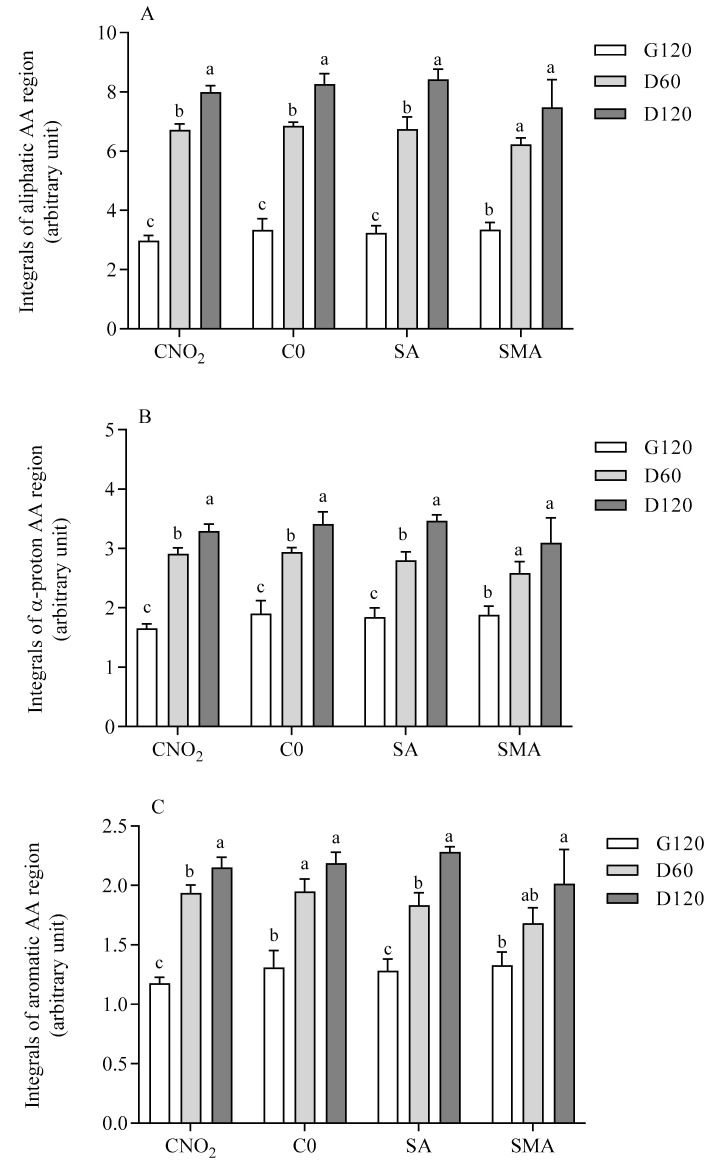
Integral area of aliphatic, α-proton, and aromatic amino acid regions at different digestion times in different salamis. Data are means ± SD of in vitro digestion of three independent samples analyzed in duplicate. Integrals of aliphatic (**A**), α-proton (**B**), and aromatic (**C**) amino acid regions are expressed as signal areas. Statistical analysis was by one-way ANOVA (always *p* < 0.05) with Tukey’s post-hoc test comparing the three digestion times in each salami formulation (different letters indicate significant differences). G120: end of gastric phase; D60: 60 min of duodenal phase; D120: end of duodenal phase; AA: amino acids; C-NO_2_: salami with sodium nitrite, potassium nitrate and with nitrate-reducing microbial starter cultures; C-0: salami containing neither nitrate-reducing microbial starter cultures nor additives (nitrite, polyphenols and ascorbate); SA: salami with nitrate-reducing microbial starter cultures and sodium ascorbate; SMA: salami with nitrate-reducing microbial starter cultures, sodium ascorbate and plant extracts.

The progression of the release of the aliphatic (A), total (B), and aromatic (C) amino acid regions at different digestion times in the four formulations of salami is shown in Figure 2, as expressed by the integral areas recorded in specific diagnostic region of the NMR spectra. The α-proton amino acid region (region B) comprises the signals of hydrogen nuclei that are present individually in all amino acids, both in the free and bound state to peptides or in soluble proteins [21]. However, only hydrogen nuclei belonging to soluble molecules generate detectable signals, thus providing direct evidence of the solubilization of fragments derived from the fibrillar insoluble proteins that are released into the digestion fluid. In each spectral region, the release of amino acids or soluble peptides from the food matrix increased over time during the digestion, with the highest release achieved with statistical significance at D120, with the exception of SMA salami, for which there was no statistical difference between D60 and D120 due to higher variance of this salami at the longest digestion time. If the aromatic region of the NMR spectra is considered for comparison, there is not statistical difference even between the gastric phase (G120) and at the middle of the duodenal phase (D60). When comparing different salami formulations at the same digestion time, integrals of the α-proton amino acid region appeared statistically lower in SMA than in C-0 during the middle of duodenal digestion (Figure S2).

### 2.4. Peptides Formation

The total number of peptides in the undigested and digested salami formulations is shown in Table 2. In all non-digested formulations, most of the identified peptides were generated from myofibrillar proteins (78%), particularly from actin (24%) and myosin VII (28%), while only 12% and 10% of the sequences came from sarcoplasmic and not-identified proteins, respectively. The number of total and myofibrillar protein-derived peptides (MF peptides) was not affected by in vitro digestion, except in C-0. In contrast, the number of peptides from sarcoplasmic proteins (SP peptides) decreased in C-NO_2_ at all times of digestion and in C-0 at G120. In all samples, peptides from not-identified proteins (NI peptides), including phosphorylase B kinase, fructose B phosphate aldolase, troponin T, myosin VI, myosin VII heavy chain, and myosin I light chain, were fully hydrolyzed during in vitro digestion, while peptides from tropomyosin α-1 chain and myosin IV were produced. The mean peptide length significantly decreased during duodenal digestion.

### 2.5. Bioinformatic Analysis

The bioactive sequences detected in undigested salami and at different time points of in vitro digestion, their semiquantitative content, and their presumed biological activity are reported in Table 3. No peptides with a documented bioactive sequence were found in undigested salami. Two bioactive sequences (AGDDAPRAVF and FQPSF) were detected in all formulations except SA (containing only FQPSF) at the end of the gastric phase, but were further hydrolyzed during the duodenal phase. Two bioactive sequences were detected in all formulations in the middle and end of duodenal digestion. Among them, at D60, AGDDAPR was more abundant in SA than in other formulations, while at D120, VAPEEHPT was more abundant in SA and C-NO_2_ (Table S3).

### 2.6. Cell Proliferation and Genotoxicity

**Figure 3:**
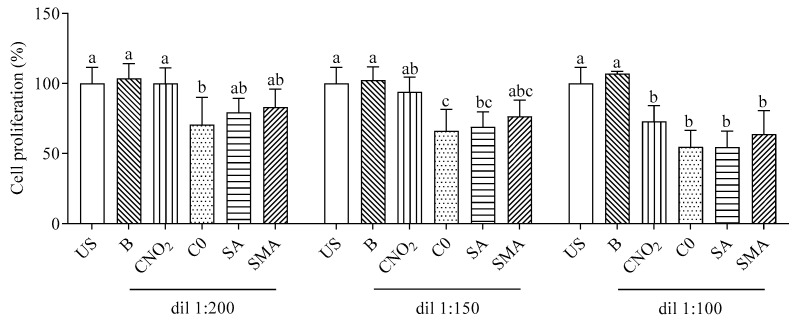
Antiproliferative effect of digested salami formulations. Data are means ± SD of three independent supplementation of in vitro digested, each one analyzed in triplicate. The antiproliferative effect is expressed as the percentage of cell numbers representing unsupplemented (US) cells (assigned 100%). Statistical analysis was by one-way ANOVA (always *p* < 0.05) with Tukey’s post-hoc test comparing US and supplemented cells for each dilution (different letters indicate significant differences). B: “blank” digestion; dil: dilution; C-NO_2_: salami with sodium nitrite, potassium nitrate and with nitrate-reducing microbial starter cultures; C-0: salami containing neither nitrate-reducing microbial starter cultures nor additives (nitrite, polyphenols and ascorbate); SA: salami with nitrate-reducing microbial starter cultures and sodium ascorbate; SMA: salami with nitrate-reducing microbial starter cultures, sodium ascorbate and plant extracts.

The antiproliferative effect of the scalar dilution of the digested salami formulations (D120) is shown in Figure 3. To avoid bias due to the antiproliferative effect of the digestion fluids, some cells (B) were supplemented with blank digestion samples at the same dilution. The inhibition of cell proliferation by digested salami was concentration-dependent. Compared to unsupplemented cells, at the lowest concentration used (dilution 1:200) only C-0 showed a significant inhibitory effect; when increasing the concentration (dilution 1:150), for SA a significant antiproliferative effect was evident. At the maximum concentration used (dilution 1:100), supplementation of all digested salami formulations caused a decrease in cell proliferation. Blank supplementation caused no effect at any dilution used.

**Figure 4:**
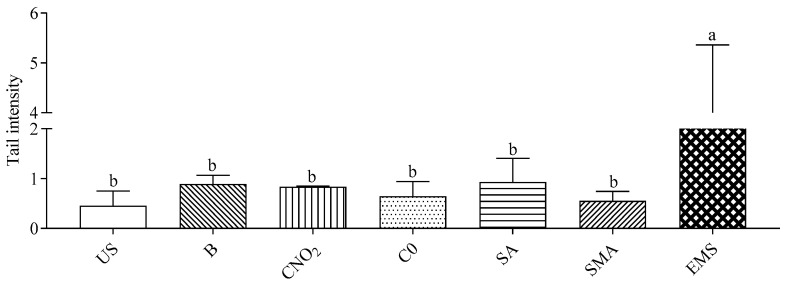
Genotoxic effect of different salami formulations. Data are means ± SD of three independent supplementation of in vitro digested, each one analyzed in triplicate. The genotoxicity is expressed as tail intensity. Statistical analysis was by one-way ANOVA (*p* < 0.05) with Tukey’s post-hoc test comparing unsupplemented (US), 2 mM ethylmethanesulfonate (EMS) and digested supplemented cells (different letters indicate significant differences). B: “blank” digestion; C-NO_2_: salami with sodium nitrite, potassium nitrate and with nitrate-reducing microbial starter cultures; C-0: salami containing neither nitrate-reducing microbial starter cultures nor additives (nitrite, polyphenols and ascorbate); SA: salami with nitrate-reducing microbial starter cultures and sodium ascorbate; SMA: salami with nitrate-reducing microbial starter cultures, sodium ascorbate and plant extracts.

The genotoxicity of the digested salami formulations (D120) is shown in Figure 4. The dilution factor (1:150) for analysis was chosen based on the reduction of about 30% of the proliferation activity. Some cells were supplemented with blank digestion samples (B) (dilution 1:150) or 2 mM ethylmethanesulfonate (EMS). Compared to US cells, blank digestion samples and all salami formulations showed no genotoxicity. Conversely, EMS supplementation had a significant genotoxic effect.

## 3. Discussion

Although nitrates/nitrites are the most widely used food additive in processed meat, their excessive intake is correlated to an increased risk of gastrointestinal cancer [22]. The general concern about nitrate/nitrite dietary intake has stimulated the development of novel approaches to reduce their use in food [23].

In this study, we investigated the effects of replacing synthetic nitrates/nitrites with a combination of natural polyphenols from plant extracts, ascorbate, and nitrate-reducing microbial starter cultures containing lactic acid bacteria and nitrate-reducing, coagulase-negative Staphylocaccaceae. Plant extracts contain various compounds (e.g., phenolics, flavonoids, tannins, and saponins) showing strong antimicrobial activity [24] and preventing lipid peroxidation [25]. Ascorbate plays a part in nitrite reduction and NO formation and it is one of the effective inhibitors in nitrosamine formation [26]. Coagulase-negative *Staphylococci* possess a nitrate-reducing activity that allows the generation of nitrite and NO generation, also favoring the formation of color in meat products without added nitrites/nitrates [27,28,29]. To allow the replacement of synthetic nitrates/nitrites, a different process was adopted for salami production.

Since formulation and processing can impact on both bioaccessibility and the release/synthesis of bioactive compounds [30,31], and the nutritional value of foods is determined not only by the chemical composition but also by the bioaccessibility of nutrients and the formation of bioactive compounds during digestion [17], we evaluated the bioaccessibility of fatty acids, the release of protein and the formation of peptides during in vitro digestion.

The different formulation/processing slightly modulated the release kinetic of fatty acid from the food matrix, and the bioaccessibility of fatty acids was similar in all salamis at the end of in vitro digestion. Although Navarro et al. [32] already showed that nitrite and nitrate per se do not affect the endogenous hydrolysis of the lipids during the fermentation process used to produce dry sausage and Pateiro et al. [33] showed that antioxidants from plant extracts increase the release of fatty acids in Chorizo dry-cured sausage, to the best of our knowledge this is the first study comparing the impact of different formulations and processing on the bioaccessibility of fatty acids during in vitro digestion.

To evaluate protein hydrolysis during in vitro digestion, three complementary spectrophotometric methods were used, which selectively quantify protein concentration with different molecular masses [20,21]. Furthermore, the method based on the NMR spectroscopy also added information on the size and solubility of the digested proteins, being sensitive to all molecules, regardless of their size, which are present in solution. The shape of the signals is informative of the molecular size, the signals belonging to fragments above 20 kDa being much larger than those belonging to smaller fragment or peptides. The simultaneous use of the four methods allowed us to hypothesize that most proteins were hydrolyzed into fragments between 3 KDa and peptides > five amino acids, which are only revealed by the UV assay, at the end of the duodenal phase without any significant difference between the four types of salami. However, a different kinetic of proteolysis was shown by analyzing with OPA and Coomassie blue assays. Indeed, at G120 the OPA assay evidenced a different extent of digestion, especially between CNO_2_ and SMA, while at D60 the same differences were shown by Coomassie blue. Absorbance at 280 nm is indicative of greater variability in the digestion process within each type of salami, highlighting a different amino acid composition, reflected in the amount of aromatic amino acids detected at 280 nm, between the different fragments released during digestion. Furthermore, differences in the kinetic of proteolysis might be related to the degree of protein oxidation [34], to the modulation of enzymatic activity by phenolic compounds [35], as well as to the food matrix effect [36].

The HR-NMR spectroscopic analysis in the digested sample was conducted to provide qualitative and quantitative information on amino acids and their oligo-/polymer structure such as solute concentration, type of functional groups, and size of the flexibility of the molecules to which the atom is bound [37]. The evident increase in the area of NMR signals during digestion confirmed the formation of hydrolyzed soluble fragments from insoluble proteins, such as myofibrillar ones. It is worth noting here that myofibrillar proteins are not soluble, therefore not detectable by NMR, but only the soluble fragments originate signals visible in the spectra, with each amino acid providing signals in different regions of the spectrum depending on the functional group in which it is bound (aromatic chain, aliphatic moieties or proximity to hydroxyl or charged groups). Furthermore, the simultaneous co-existence of both narrow and wide signals in the spectrum (not shown) confirmed the simultaneous presence of small peptides and larger fragments with a molecular mass compatible with what was detected by spectrophotometric analysis. This trend was confirmed by the analysis of peptides after in vitro digestion, which gave rise to sequences with different lengths and molecular weights in the three stages of digestion, ranging from an average number of 11 amino acids in the samples before digestion and at the end of the gastric phase, to an average value of 6 amino acids at the end of the duodenal digestion.

Although no peptides with an already reported bioactivity were identified in undigested samples, new bioactive sequences were released during digestion. More specifically, a peptide (VAPEEHPT) generated by the cleavage of intact actin and identified as bioactive was released midway through the duodenal phase and found intact at the end of digestion. This peptide, recognized as a dipeptidyl peptidase (DPP)-IV inhibitor fragment, was also found by Paolella et al. in dry-cured ham digesta [38], and it is encrypted in the longer sequence LRVAPEEHPTL already identified in beef and trout digesta [39]. These sequences contain the bioactive tripeptide VAP, which has been extensively associated with ACE inhibitory activity [39,40,41]. VAPEEHPT carries Val and Ala at the N-terminus, a feature playing an important role in ACE inhibition [42] and associated with an increased antioxidant activity compared to peptides with a prevalence of hydrophilic residues [43]. At D60, the VAPEEHPT sequence was present in the same amount in all salami formulations, while it was significantly more abundant in SA and C-NO_2_ at the end of the duodenal digestion.

The aromatic peptide AGDDAPRAVF, released during the gastric phase and identified in Spanish, Belgian and Italian dry-fermented sausages [44], was further hydrolyzed during duodenal digestion into the antioxidant, lipase and α-amylase inhibitor sequence AGDDAPR. Both AGDDAPRAVF and AGDDAPR encrypt in their sequence the AG dipeptide, known for its ACE inhibitory activity [44]. At D60, the AGDDAPR sequence was significantly more abundant in SA, while no significant differences were found among the different salami formulations at the end of duodenal digestion (D120).

It should be noted that all bioactive sequences detected contained proline, a feature related to increased resistance to gastrointestinal enzymes and which requires the action of proline-specific peptidases [45].

Since nitrates/nitrites are linked to toxic effects in intestinal cells [46,47,48], the cytotoxicity and genotoxicity of the different formulations were evaluated in the HT-29 cell line. To avoid misleading results, the potential cytotoxicity of digested salami was evaluated prior to genotoxic experiments. As previously reported [49], supplementation of digested samples to cultured cells caused a concentration-dependent cytotoxic effect. Although there is evidence of a connection between nitrate and nitrite intake and a higher relative risk of different types of cancer [4,50], the salami formulation including these additives showed cytotoxicity only at the lower dilution used (1:100), and no genotoxic effect. This could be explained by the lack of conversion of nitrates to ammonia by enteric bacteria, which normally occurs in vivo. Indeed, several enteric bacteria have shown the ability of catalytic reduction of nitrates to ammonia via nitrites during dissimilatory respiration [51], and chronic exposure to ammonia has been associated with oxidative stress, inflammation, and disbalance of microtubule activity and nutrient transporters in intestinal cells [52]. Before drawing any conclusion, a platform for co-culture of cell lines with anaerobic probiotic bacteria should be mandatory to study their biological effect in the gut.

## 4. Materials and Methods

### 4.1. Materials

Unless specified, chemicals and solvents were of the highest analytical grade and purchased from Merck (Darmstadt, Germany) and Sigma-Aldrich (St. Louis, MO, USA).

### 4.2. Salami Formulation, Preparation, and Fermentation

Salamis were manufactured at the Stazione Sperimentale per l’Industria delle Conserve Alimentari (SSICA, Italy). Four different salami formulations were tested. For all the formulations, the salami mixture consisted of lean muscle tissue (75%) and minced bacon (25%). The meat was weighed, cut into small pieces, ground in a meat mincer (∅ = 6 mm plate), and then mixed with salt (2.5%), dextrose (0.2%), ascorbate (0.05%) and natural flavors.

The positive control formulation (C-NO_2_) was added with sodium nitrite, potassium nitrate and nitrate-reducing microbial starter cultures (MSC). MSC (Chr. Hansen, S.p.A., Parma, Italy) contained lactic acid bacteria and nitrate-reducing coagulase negative *staphylocaccaceae*, and was inoculated using common manufacturing practices to properly drive the fermentation phase and to promote the development of aroma during the ripening phase.

Two innovative formulations not containing nitrites were prepared: the first (SA) was added with MSC and sodium ascorbate (0.3%); the second (SMA) was added with MSC, sodium ascorbate (0.3%) and plant extracts from grapeseed, green tea and olive (Indena S.p.A., Milan, Italy), characterized according to their total polyphenols and nitrate content to provide 0.4 g/kg of bioactive polyphenols and less than 1 ppm of total nitrate to the meat mixture. Finally, the “negative control” (C-0) was prepared with neither MSC nor additives (nitrite, polyphenols and ascorbate).

The salami formulations were prepared in a mixer and stuffed into natural casings separately (∅ = 55 mm) to obtain salamis with an average weight of 470 ± 25 g. The recipe for the preparation of the salami batter is reported in Table 4. The positive controls (C-NO_2_) were subjected to a traditional hot-drying method, while the nitrite-free formulations were subjected to cold-drying until the water activity and pH were low enough to avoid microbiological risk, then they were conventionally ripened. Ripening was conducted in a temperature and atmosphere-controlled climate camera at 13–15 °C, with a relative humidity of 75–83%, and ended when weight loss of a 38–40% was achieved.

Each salami formulation was prepared in triplicate in three different days. A detailed description of salami processing has been recently reported [53].

The consumer test performed at SSICA did not reveal any differences in terms of color, texture and rancid flavor between control and experimental salami, without negative sensory properties induced by the plant extracts.

### 4.3. In Vitro Digestion

According to the standardized INFOGEST protocol [54], the digestion process was performed on 45 g of salami for 242 min (2 min of oral, 120 min of gastric and 120 min of intestinal digestion) at 37 °C. To simulate chewing, salami formulations were chopped before starting oral digestion. During the process, several consecutive enzymatic reactions took place by the addition of simulated saliva (15.1 mM KCl, 3.7 mM KH_2_PO_4_, 13.6 mM NaHCO_3_, 0.15 mM MgCl_2_(H_2_O)_6_, 0.06 mM (NH_4_)_2_CO_3_, 0.75 mM CaCl_2_ pH 7), simulated gastric juice (6.9 mM KCl, 0.9 mM KH_2_PO_4_, 25 mM NaHCO_3_, 47.2 mM NaCl, 0.12 mM MgCl_2_(H_2_O)_6_, 0.5 mM (NH_4_)_2_CO_3_, 75 µM CaCl_2_ containing 2,000 U/mL pepsin) at pH 3, and simulated pancreatic juice (6.8 mM KCl, 0.8 mM KH_2_PO_4_, 85 mM NaHCO_3_, 38.4 mM NaCl, 0.33 mM MgCl_2_(H_2_O)_6_, 0.3 mM CaCl_2_ containing 10 mM bile and 100 U/mL pancreatin) at pH 7. Samples were taken at the end of the gastric phase (G120), after 60 min (D60) and at the end of the duodenal phase (D120). In G120 samples, the pH was increased to 7 with 35% NaOH to stop the pepsin hydrolytic action and reported to 3 with 37% HCl. Samples at D60 and D120 were acidified to pH 3 with 37% HCl to stop pancreatic hydrolysis and reported to 7 with 35% NaOH. Digested samples were centrifuged at 50,000× *g* for 15 min and the surfaced upper oil phase discarded. To remove any turbidity, the supernatant consisting of the aqueous micellar phases was filtered with 0.2 μm cellulose acetate membranes and stored at −80 °C until further analysis. In each salami formulation, digestion was performed in triplicate and triplicates were then combined.

### 4.4. Fatty Acids Bioaccessibility

Total lipids were extracted according to Folch et al. [55]. After methyl-esterification [56], the quantitative and qualitative content of fatty acid methyl esters (FAMEs) was determined by fast-GC (GC-2030AF; Shimadzu, Kyoto, Japan) using a capillary column (30 mt, 0.2 μm film thickness) with a programmed temperature gradient (50–250 °C, 10 °C/min). The gas chromatographic peaks were identified using authentic samples based on their retention time. FAMEs from chemicals added during in vitro digestion were subtracted and quantitative evaluations were normalized for the dilution factor due to the addition of digestive fluids. Bioaccessibility was assessed as FAME content in digested sample/FAME content in the corresponding sample before digestion × 100 [57].

### 4.5. Protein Hydrolysis

In undigested salami, protein content was determined by the Kjeldahl method [58]. In digested samples, protein/peptide concentration was determined spectrophotometrically by three different methods, namely, measuring the absorbance at 280 nm [17], the Coomassie assay [59], and the o-phthaldialdehyde (OPA) assay [60], using non-fatty dry milk, bovine serum albumin, and L-isoleucine as a standard, respectively. Protein content from enzymes added during in vitro digestion was subtracted and values were normalized for the dilution factor due to the addition of digestive fluids.

### 4.6. HR ^1^H NMR Spectroscopy

Digested samples were centrifuged at 2300× *g* for 5 min at 4 °C to eliminate any particulate matter formed during freezing/thawing and then 750 μL of supernatant was taken and added to 120 μL of 100 mM phosphate buffer with 10 mM trimethylsilylpropanoic acid (TSP). HR-NMR analysis was recorded at 298 K on a Bruker US+ Avance III spectrometer (Bruker, Billerica, MA, USA) operating at a proton frequency of 600.13 MHz as previously reported [17].

### 4.7. Bioactive Peptides Determination and Identification

Digested samples were centrifuged at 14,000 rpm for 40 min at 4 °C to remove any particulate matter formed during freezing/thawing. The supernatants were filtered through 0.22 µm PTFE filters and directly injected into the LC–HRMS system, as previously reported [17]. The software used for data processing was UNIFI (Waters Corporation, Milford, MA, USA). The following protein Uniprot accession numbers were employed in processing: P68137 (actin), Q9TV61 (myosin-1), Q9TV62 (myosin-4), Q9TV63 (myosin-2), P79293 (myosin-7), F2Z5B6 (tropomyosin alpha-1 chain), P08835 (albumin), P02189 (myoglobin), and F1SHL9 (pyruvate kinase). A minimum threshold of three amino acids was set in the processing parameters. Variable amino acid modifications were included as deamidation (N, Q) pyroglutamic acid N-term (E, Q), oxidation (single or double, M or W), phosphorylation (S, T, Y). Peptide semiquantitative data were obtained as normalized areas. Released bioactive sequences were identified using a bioinformatic approach.

The whole set of peptide sequences under analysis was searched for in two benchmark databases of peptide bioactivity, namely BIOPEP (http://www.uwm.edu.pl/biochemia/index.php/en/biopep, accessed on 4 July 2022) [61] and AHTPDB (http://crdd.osdd.net/raghava/ahtpdb/, accessed on 4 July 2022) [62], which were queried automatically and systematically employing a scripted pipeline developed «in-house».

### 4.8. HT-29 Cell Culture and Supplementation

The human colon adenocarcinoma cell line HT-29 was kindly obtained from the Northern Ireland Centre for Food and Health. Cells were grown in DMEM added with 100 U/mL penicillin, 100 μg/mL streptomycin, 2 mM L-glutamine and 10% fetal bovine serum, in a humidified CO_2_ (5%) incubator at 37 °C. For antiproliferative experiments, cells were seeded at 5 × 10^3^ cells/well into 96-well plates and supplemented with scalar concentrations (1:200–1:100) of D120 digested samples in free serum DMEM for 24 h. For genotoxic experiments, cells were supplemented with a 1:150 concentration of D120 digested samples or 2 mM ethylmethanesulfonate (EMS) in free serum DMEM for 24 h. To avoid interference, in each experiment some cells (B) received the same dilution of the solution obtained from a ‘blank’ digestion, comprising an in vitro digestion performed without the addition of any food.

### 4.9. Cell Proliferation

Anti-proliferative effect was evaluated by using CellTiter 96^®^ AQueous One Solution Cell Proliferation Assay (Promega Corporation, Madison, WI, USA), following the manufacturer’s instructions. After treatment, 3-(4,5-dimethylthiazol-2-yl)-5-(3-carboxymethoxyphenyl)-2-(4-sulfophenyl)-2H-tetrazolium salt (MTS) was added to each well and the absorbance of new soluble formazan product in DMEM was measured after 4 h at 485 nm by a Tecan SpectraFluor Plus plate reader (Tecan, Männedorf, Switzerland). Cell viability was expressed as a percentage with respect to unsupplemented cells, assigned as 100%.

### 4.10. Genotoxicity Assay

Genotoxic effect was evaluated by alkaline single-cell gel electrophoresis (Comet Assay) [63], with minor modifications. After supplementation, cells were washed twice with PBS and trypsinized. Cells were resuspended in 90 μL of low melting 0.7% agarose and transferred onto degreased microscope slides previously dipped in 1% normal melting agarose for the first layer and covered with a third layer of low melting 0.7% agarose. Cell lysis was carried out overnight at 4 °C by a lysis buffer (2.5 M NaCl, 100 mM EDTA, 8 mM Tris–HCl, 1% Triton X-100 and 10% dimethyl sulfoxide, pH 10). The electrophoretic migration (0.78 V/cm, 300 mA for 20 min) was performed in an alkaline buffer (1 mM EDTA, 300 mM NaOH, 0 °C, pH > 13). DNA was stained with 75 μL ethidium bromide (10 μg/mL) before examination at 100× magnification under a Leica DMLS fluorescence microscope (excitation filter BP 515–560 nm, barrier filter LP 580 nm), using an automatic image analysis system (Comet Assay IV—Perceptive Instruments Ltd., Bury St Edmunds, UK). The total percentage of fluorescence in the tail (TI, tail intensity) provided representative data on genotoxic effects (Figure S3). For each sample, coded and evaluated blind, 100 cells were analyzed, and the median value of TI was calculated.

### 4.11. Statistical Analysis

Statistical differences were evaluated by the one-way analysis of variance (ANOVA) followed by Tukey’s post hoc test using Prism software ver. 7.0 (GraphPad, San Diego, CA, USA). Different letters indicate significant differences (at least *p* < 0.05).

## 5. Conclusions

Based on current results, the replacement of synthetic nitrites and nitrates with nitrate-reducing microbial starter cultures, along with the addition of ascorbate and natural antioxidants from plant sources, appears to be a promising strategy to develop innovative “clean label” salami. In fact, the innovative formulation/processing did not negatively affect the release of fatty acids and the hydrolysis of proteins during digestion. Further studies are needed to evaluate in depth the organoleptic features of the developed products as well their shelf-life.

## Figures and Tables

**Table 1:** Time-course of fatty acid bioaccessibility in the different salami formulations. Data are means ± SD of in vitro digestion of three independent samples analyzed in duplicate. Fatty acid bioaccessibility is expressed as a percentage, and it was calculated as the concentration of fatty acid methyl ester in digested salami/concentration in salami before digestion × 100. Statistical analysis was by one-way ANOVA (always *p* < 0.05) with Tukey’s post-hoc test comparing each salami formulation at the three digestion time points (different letters indicate statistical significance). G120: end of gastric phase; D60: mid duodenal phase; D120: end of duodenal phase; C-NO_2_: salami with sodium nitrite, potassium nitrate and with nitrate-reducing microbial starter cultures; C-0: salami containing neither nitrate-reducing microbial starter cultures nor additives (nitrite, polyphenols and ascorbate); SA: salami with nitrate-reducing microbial starter cultures and sodium ascorbate; SMA: salami with nitrate-reducing microbial starter cultures, sodium ascorbate and plant extracts.

	CNO_2_			C0			SA			SMA		
	G120	D60	D120	G120	D60	D120	G120	D60	D120	G120	D60	D120
14:0	0.5 ± 0.7 c	30.6 ± 4.2 b	37.3 ± 0.2 a	0.3 ± 0.2 b	38.0 ± 6.5 a	43.6 ± 6.4 a	0.4 ± 0.1 b	37.7 ± 1.8 a	42.1 ± 3.1 a	0.7 ± 0.4 b	38.6 ± 2.4 a	40.0 ± 0.6 a
16:0	0.8 ± 0.4 c	29.2 ± 3.0 b	37.4 ± 2.7 a	0.4 ± 0.2 b	39.2 ± 8.3 a	45.6 ± 7.9 a	1.0 ± 0.1 c	32.3 ± 1.1 b	43.9 ± 4.2 a	0.9 ± 0.5 b	40.2 ± 2.2 a	37.5 ± 0.0 a
16:1 n−7	0.4 ± 0.5 b	27.0 ± 4.7 a	31.7 ± 0.3 a	0.0 ± 0.0 b	32.6 ± 5.7 a	37.1 ± 5.7 a	0.0 ± 0.0 b	29.8 ± 3.1 a	37.9 ± 4.8 a	0.1 ± 0.1 b	35.0 ± 2.8 a	38.2 ± 2.5 a
18:0	0.9 ± 0.6 b	28.2 ± 1.2 a	38.4 ± 8.5 a	0.6 ± 0.1 b	40.2 ± 8.9 a	47.3 ± 0.8 a	1.2 ± 0.1 c	30.8 ± 1.0 b	43.5 ± 4.6 a	1.0 ± 0.3 c	30.4 ± 2.2 b	40.3 ± 1.8 a
18:1 n−9	0.6 ± 0.4 b	31.0 ± 4.7 a	37.5 ± 0.6 a	0.2 ± 0.1 b	37.2 ± 7.9 a	43.1 ± 6.6 a	0.8 ± 0.0 c	33.0 ± 2.7 b	42.3 ± 4.0 a	0.6 ± 0.5 c	39.0 ± 2.4 b	41.8 ± 2.8 a
18:2 n−6	0.6 ± 0.5 c	36.6 ± 3.8 b	45.2 ± 3.0 a	0.3 ± 0.1 b	39.9 ± 8.7 a	47.2 ± 6.3 a	0.4 ± 0.1 c	42.4 ± 5.4 b	54.4 ± 6.2 a	0.6 ± 0.4 b	44.0 ± 3.5 a	48.1 ± 2.1 a
18:3 n−3	0.0 ± 0 b	40.3 ± 14.0 a	45.2 ± 4.5 a	0.6 ± 1.1 b	38.7 ± 19.7 a	51.2 ± 6.1 a	0.0 ± 0.0 b	41.2 ± 8.1 a	51.2 ± 4.1 a	0.0 ± 0.0 b	44.3 ± 6.7 a	49.8 ± 1.1 a
20:1 n−9	0.0 ± 0 b	22.1 ± 7.5 a	29.3 ± 0.8 a	0.0 ± 0 b	30.1 ± 7.3 a	38.1 ± 5.1 a	0.0 ± 0.0 b	25.0 ± 2.5 a	35.6 ± 5.8 a	0.0 ± 0.0 b	30.4 ± 0.8 a	33.4 ± 3.6 a
20:4 n−6	0.0 ± 0 b	46.3 ± 3.2 a	70.3 ± 17.3 a	0.0 ± 0 b	53.0 ± 16.7 a	70.0 ± 14.4 a	0.0 ± 0.0 b	49.5 ± 12.3 a	70.3 ± 14.6 a	0.0 ± 0.0 c	45.0 ± 2.3 b	50.8 ± 3.2 a
Total	0.6 ± 0.4 c	30.7 ± 3.8 b	38.3 ± 2.5 a	0.33 ± 0.1 b	38.3 ± 8.2 a	44.7 ± 7.4 a	0.8 ± 0.0 c	33.3 ± 1.3 b	44.1 ± 3.6 a	0.7 ± 0.4 b	39.9 ± 2.4 a	39.8 ± 1.3 a

**Table 2:** Number of peptides in non-digested and digested salami at different digestion time. Data are means ± SD of non-digested or in vitro digestion of three independent samples analyzed in duplicate. Statistical analysis was performed a by one-way ANOVA with Tukey’s post-hoc test comparing each salami before digestion and at the three digestion times (different letters indicate significant differences). G120: end of gastric phase; D60: 60 min of duodenal phase; D120: end of duodenal phase. MF: myofibrillar; SP: sarcoplasmic; HC: heavy chain; LC: light chain; PK: pyruvate kinase; FBA: fructose bisphosphate aldolase; PBK: phosphorylase B kinase; NI: not identified; ND: not digested, AA: aminoacids; n.s.: not significant; C-NO_2_: salami with sodium nitrite, potassium nitrate and with nitrate-reducing microbial starter cultures; C-0: salami containing neither nitrate-reducing microbial starter cultures nor additives (nitrite, polyphenols and ascorbate); SA: salami with nitrate-reducing microbial starter cultures and sodium ascorbate; SMA: salami with nitrate-reducing microbial starter cultures, sodium ascorbate and plant extracts.

Protein Source (UNIPROT)	Type	CNO_2_				ANOVA	C0				ANOVA
		ND	G120	D60	D120	*p* Value	ND	G120	D60	D120	*p* Value
Actin (P68137)	MF	12.0 ± 0.0 a	25.5 ± 6.4 a	10.0 ± 4.2 a	11.5 ± 0.7 a	<0.05	12.0 ± 0 b	22.5 ± 0.7 a	9.5 ± 2.1 b	14.0 ± 1.4 b	<0.05
Myosin I (Q9TV61)	MF	4.0 ± 0.0 a	0.5 ± 0.7 a	1.5 ± 2.1 a	2.0 ± 1.4 a	n.s.	4.0 ± 0 a	2.5 ± 0.7 ab	0.5 ± 0.7 b	2.5 ± 0.7 b	<0.05
Myosin II (Q9TV63)	MF	1.0 ± 0.0 a	0.0 ± 0.0 a	0.5 ± 0.7 a	0.5 ± 0.7 a	n.s.	1.0 ± 0 a	0.0 ± 0.0 a	0.5 ± 0.7 a	1.0 ± 0.0 a	n.s.
Myosin IV (Q9TV62)	MF	0.0 ± 0.0 a	3.0 ± 1.4 a	7.0 ± 4.2 a	6.5 ± 2.1 a	n.s.	0.0 ± 0.0 b	3.5 ± 0.7 b	4.5 ± 2.1 b	11.0 ± 1.4 a	<0.05
Myosin VI (Q29122)	MF	3.0 ± 0.0 a	0.0 ± 0.0 b	0.0 ± 0.0 b	0.0 ± 0.0 b	<0.05	3.0 ± 0 a	0.0 ± 0.0 b	0.0 ± 0.0 b	0.0 ± 0.0 b	<0.05
Myosin VII (P79293)	MF	14.0 ± 0.0 a	1.0 ± 0 a	11.0 ± 4.2 a	13.5 ± 6.4 a	n.s.	14.0 ± 0 a	3.0 ± 1.4 c	8.5 ± 2.1 b	19.0 ± 0 a	<0.05
Myosin I LC (A1XQT6)	MF	3.0 ± 0.0 a	0.0 ± 0.0 b	0.0 ± 0.0 b	0.0 ± 0.0 b	<0.05	3.0 ± 0 a	0.0 ± 0.0 b	0.0 ± 0.0 b	0.0 ± 0.0 b	<0.05
Myosin VII HC (K7GMH0)	MF	1.0 ± 0.0 a	0.0 ± 0.0 b	0.0 ± 0.0 b	0.0 ± 0.0 b	<0.05	1.0 ± 0 a	0.0 ± 0.0 b	0.0 ± 0.0 b	0.0 ± 0.0 b	<0.05
Tropomyosin α-1 chain (F2Z5B6)	MF	0.0 ± 0.0 c	0.0 ± 0.0 c	5.0 ± 0 a	3.5 ± 0.7 b	<0.05	0.0 ± 0.0 a	0.0 ± 0.0 a	3.0 ± 1.4 a	4.5 ± 0.7 a	<0.05
Troponin T (Q75NG6)	MF	1.0 ± 0 a	0 ± 0 b	0.0 ± 0.0 b	0.0 ± 0.0 b	<0.05	1.0 ± 0.0 a	0.0 ± 0.0 b	0.0 ± 0.0 b	0.0 ± 0.0 b	<0.05
PK (F1SHL9)	SP	3.0 ± 0 a	1.0 ± 0 c	3.0 ± 0 a	2.0 ± 0 b	<0.05	3.0 ± 0.0 a	1.0 ± 0 a	4.0 ± 2.8 a	3.5 ± 0.7 a	n.s.
Albumin (P008835)	SP	1.0 ± 0.0 a	0.5 ± 0.7 a	0.5 ± 0.7 a	1.0 ± 0 a	n.s.	1.0 ± 0 a	0 ± 0 a	0.5 ± 0.7 a	0.5 ± 0.7 a	n.s.
FBA (F1RJ25)	SP	1.0 ± 0.0 a	0.0 ± 0.0 b	0.0 ± 0.0 b	0 ± 0 b	<0.05	1.0 ± 0 a	0 ± 0 b	0 ± 0 b	0.0 ± 0.0 b	<0.05
PBK (F1RP07)	SP	1.0 ± 0.0 a	0.0 ± 0.0 b	0.0 ± 0.0 b	0.0 ± 0.0 b	< 0.05	1.0 ± 0.0 a	0.0 ± 0.0 b	0.0 ± 0.0 b	0.0 ± 0.0 b	<0.05
NI		5.0 ± 0.0 a	0.0 ± 0.0 b	0.0 ± 0.0 b	0.0 ± 0.0 b	< 0.05	5.0 ± 0.0 a	0.0 ± 0.0 b	0.0 ± 0.0 b	0.0 ± 0.0 b	<0.05
MF peptides		39.0 ± 0.0 a	30.0 ± 5.7 a	35.0 ± 15.6 a	37.5 ± 6.4 a	n.s.	39.0 ± 0.0 a	31.5 ± 3.5 a	26.5 ± 9.2 b	52.0 ± 4.2 a	n.s.
SP peptides		6.0 ± 0.0 a	1.5 ± 0.7 b	3.5 ± 0.7 b	3.0 ± 0.0 b	< 0.05	6.0 ± 0.0 a	1.0 ± 0.0 b	4.5 ± 2.1 ab	4.0 ± 0.0 ab	<0.05
Total peptides		50.0 ± 0.0 a	31.5 ± 4.9 a	38.5 ± 16.3 a	40.5 ± 6.4 a	n.s.	50.0 ± 0.0 ab	32.5 ± 3.5 bc	31.0 ± 7.1 c	56.0 ± 4.2 a	<0.05
Average peptides lengths (in AA)		11.0 ± 0.0 a	12.0 ± 0.0 a	6.0 ± 0.0 b	6.0 ± 0.0 b	<0.05	11.0 ± 0.0 a	12.5 ± 0.7 a	6.0 ± 0.0 b	6.0 ± 0.0 b	<0.05
Protein source (UNIPROT)	Type	SA				ANOVA	SMA				ANOVA
		ND	G120	D60	D120	*p* value	ND	G120	D60	D120	*p* value
Actin (P68137)	MF	12.0 ± 0.0 b	23.5 ± 3.5 a	10.0 ± 1.4 b	11.0 ± 1.4 b	<0.05	12.0 ± 0.0 b	23.0 ± 1.4 a	12.5 ± 2.1 b	14.0 ± 0.0 b	<0.05
Myosin I (Q9TV61)	MF	4.0 ± 0.0 a	0.5 ± 0.7 a	1.0 ± 1.4 a	1.0 ± 1.4 a	n.s.	4.0 ± 0.0 a	2.0 ± 0.0 a	1.0 ± 1.4 a	1.5 ± 2.1 a	n.s.
Myosin II (Q9TV63)	MF	1.0 ± 0.0 a	0.5 ± 0.7 a	0.5 ± 0.7 a	1.0 ± 0.0 a	n.s.	1.0 ± 0.0 a	0.0 ± 0.0 b	1.0 ± 0.0 a	1.0 ± 0.0 a	<0.05
Myosin IV (Q9TV62)	MF	0.0 ± 0.0 c	2.5 ± 0.7 bc	7.0 ± 0.0 a	5.0 ± 1.4 ab	<0.05	0.0 ± 0.0 a	2.5 ± 0.7 a	6.0 ± 1.4 a	8.5 ± 4.9 a	n.s.
Myosin VI (Q29122)	MF	3.0 ± 0.0 a	0.0 ± 0.0 b	0.0 ± 0.0 b	0.0 ± 0.0 b	<0.05	3.0 ± 0.0 a	0.0 ± 0.0 b	0.0 ± 0.0 b	0.0 ± 0.0 b	<0.05
Myosin VII (P79293)	MF	14.0 ± 0.0 ab	0.5 ± 0.7 b	11.5 ± 6.4 ab	15.5 ± 3.5 ab	<0.05	14.0 ± 0.0 a	1.5 ± 0.7 b	12.0 ± 0.0 a	15.0 ± 4.2 a	<0.05
Myosin I LC (A1XQT6)	MF	3.0 ± 0.0 a	0.0 ± 0.0 b	0.0 ± 0.0 b	0.0 ± 0.0 b	<0.05	3.0 ± 0.0 a	0.0 ± 0.0 b	0.0 ± 0.0 b	0.0 ± 0.0 b	<0.05
Myosin VII HC (K7GMH0)	MF	1.0 ± 0.0 a	0.0 ± 0.0 b	0.0 ± 0.0 b	0.0 ± 0.0 b	<0.05	1.0 ± 0.0 a	0.0 ± 0.0 b	0.0 ± 0.0 b	0.0 ± 0.0 b	<0.05
Tropomyosin α-1 chain (F2Z5B6)	MF	0.0 ± 0.0 a	0.0 ± 0.0 a	3.0 ± 2.8 a	4.5 ± 2.1 a	n.s.	0.0 ± 0.0 b	0.0 ± 0.0 b	4.0 ± 1.4 a	3.5 ± 0.7 a	<0.05
Troponin T (Q75NG6)	MF	1.0 ± 0.0 a	0.0 ± 0.0 b	0.0 ± 0.0 b	0.0 ± 0.0 b	<0.05	1.0 ± 0.0 a	0.0 ± 0.0 b	0.0 ± 0.0 b	0.0 ± 0.0 b	<0.05
PK (F1SHL9)	SP	3.0 ± 0.0 a	1.5 ± 0.7 a	1.0 ± 0.0 a	4.5 ± 2.1 a	n.s.	3.0 ± 0.0 a	3.0 ± 1.4 a	4.0 ± 1.4 a	4.0 ± 1.4 a	n.s.
Albumin (P008835)	SP	1.0 ± 0.0 a	0.5 ± 0.7 a	1.0 ± 0.0 a	0.5 ± 0.7 a	n.s.	1.0 ± 0.0 a	1.0 ± 0.0 a	0.5 ± 0.7 a	0.5 ± 0.7 a	n.s.
FBA (F1RJ25)	SP	1.0 ± 0.0 a	0.0 ± 0.0 b	0.0 ± 0.0 b	0.0 ± 0.0 b	<0.05	1.0 ± 0.0 a	0.0 ± 0.0 b	0.0 ± 0.0 b	0.0 ± 0.0 b	<0.05
PBK (F1RP07)	SP	1.0 ± 0.0 a	0.0 ± 0.0 b	0.0 ± 0.0 b	0.0 ± 0.0 b	<0.05	1.0 ± 0.0 a	0.0 ± 0.0 b	0.0 ± 0.0 b	0.0 ± 0.0 b	<0.05
NI		5.0 ± 0.0 a	0.0 ± 0.0 b	0.0 ± 0.0 b	0.0 ± 0.0 b	<0.05	5.0 ± 0.0 a	0.0 ± 0.0 b	0.0 ± 0.0 b	0.0 ± 0.0 b	<0.05
MF peptides		39.0 ± 0.0 a	27.5 ± 2.1 a	33.0 ± 12.7 a	38.0 ± 9.9 a	n.s.	39.0 ± 0.0 a	29.0 ± 0.0 a	36.5 ± 2.1 a	43.5 ± 12.0 a	n.s.
SP peptides		6.0 ± 0.0 a	2.0 ± 0.0 a	2.0 ± 0.0 a	5.5 ± 2.1 a	<0.05	6.0 ± 0.0 a	4.0 ± 1.4 a	4.5 ± 0.7 a	4.5 ± 0.0 a	n.s.
Total peptides		50.0 ± 0.0 a	29.5 ± 2.1 a	35.0 ± 12.7 a	43.5 ± 7.8 a	n.s.	50.0 ± 0.0 a	33.0 ± 1.4 a	41.0 ± 1.4 a	48.0 ± 11.3 a	n.s.
Average peptides lengths (in AA)		11.0 ± 0.0 a	11.0 ± 0.0 a	6.0 ± 0.0 b	6.0 ± 0.0 b	<0.05	11.0 ± 0.0 a	11.5 ± 0.7 a	6.0 ± 0.0 b	6.0 ± 0.0 b	<0.05

**Table 3:** Relative bioactive peptide abundance in non-digested and digested salami at different digestion time points. Data are means ± SD of non-digested or in vitro digestion of three independent samples analyzed in duplicate. Statistical analysis was performed by a one-way ANOVA with Tukey’s post-hoc test comparing each salami before digestion and at the three digestion time points (different letters indicate significant differences). G120: end of gastric phase; D60: 60 min of duodenal phase; D120: end of duodenal phase ND: not digested; DPP-IV: dipeptidyl peptidase 4; ACE: angiotensin-converting enzyme; n.r.: not reported; C-NO_2_: salami with sodium nitrite, potassium nitrate and with nitrate-reducing microbial starter cultures; C-0: salami containing neither nitrate-reducing microbial starter cultures nor additives (nitrite, polyphenols and ascorbate); SA: salami with nitrate-reducing microbial starter cultures and sodium ascorbate; SMA: salami with nitrate-reducing microbial starter cultures, sodium ascorbate and plant extracts.

Protein Sequence	Protein Source	CNO_2_				ANOVA *p* Value	C0				ANOVA *p* Value	Reported Activity (µM IC_50_)
		ND	G120	D60	D120		ND	G120	D60	D120		
FQPSF	Actin (P68137)	0.0 ± 0.0 b	2.9 ± 0.5 a	0.0 ± 0.0 b	0.0 ± 0.0 b	<0.05	0.0 ± 0.0 b	2.7 ± 0.2 a	0.0 ± 0.0 b	0.0 ± 0.0 b	<0.05	ACE inhibitor (12.6)
AGDDAPRAVF	Actin (P68137)	0.0 ± 0.0 b	2.6 ± 0.3 a	0.0 ± 0.0 b	0.0 ± 0.0 b	<0.05	0.0 ± 0.0 b	2.9 ± 0.1 a	0.0 ± 0.0 b	0.0 ± 0.0 b	<0.05	Bitterness suppressing (n.r.)
AGDDAPR	Actin (P68137)	0.0 ± 0.0 c	0.0 ± 0.0 c	2.7 ± 0.0 b	3.1 ± 0.2 a	<0.05	0.0 ± 0.0 c	0.0 ± 0.0 c	3.8 ± 0.0 a	2.9 ± 0.3 b	<0.05	Antioxidant (n.r.), ACE (11.9), pancreatic lipase (110.6), and α-amylase (14.7) inhibitor
VAPEEHPT	Actin (P68137)	0.0 ± 0.0 b	0.0 ± 0.0 b	3.0 ± 0.3 a	3.1 ± 0.3 a	<0.05	0.0 ± 0.0 c	0.0 ± 0.0 c	3.7 ± 0.5 a	2.7 ± 0.0 b	<0.05	DPP-IV inhibitor (n.r.)
Protein sequence	Protein source	SA				ANOVA *p* value	SMA				ANOVA *p* value	Reported activity (µM IC_50_)
		ND	G120	D60	D120		ND	G120	D60	D120		
FQPSF	Actin (P68137)	0.0 ± 0.0 b	3.4 ± 0.1 a	0.0 ± 0.0 b	0.0 ± 0.0 b	<0.05	0.0 ± 0.0 b	3.1 ± 0.1 a	0.0 ± 0.0 b	0.0 ± 0.0 b	<0.05	ACE inhibitor (12.6)
AGDDAPRAVF	Actin (P68137)	0.0 ± 0.0 a	0.0 ± 0.0 a	0.0 ± 0.0 a	0.0 ± 0.0 a	n.s.	0.0 ± 0.0 b	2.9 ± 0.0 a	0.0 ± 0.0 b	0.0 ± 0.0 b	<0.05	Bitterness suppressing (n.r.)
AGDDAPR	Actin (P68137)	0.0 ± 0.0 c	0.0 ± 0.0 c	4.3 ± 0.3 a	3.4 ± 0.1 b	<0.05	0.0 ± 0.0 c	0.0 ± 0.0 c	3.7 ± 0.0 a	2.8 ± 0.1 b	<0.05	Antioxidant (n.r.), ACE (11.9), pancreatic lipase (110.6), and α-amylase (14.7) inhibitor
VAPEEHPT	Actin (P68137)	0.0 ± 0.0 c	0.0 ± 0.0 c	3.6 ± 0.2 a	3.2 ± 0.1 b	<0.05	0.0 ± 0.0 c	0.0 ± 0.0 c	3.1 ± 0.0 a	2.7 ± 0.0 b	<0.05	DPP-IV inhibitor (n.r.)

**Table 4:** Salami recipe.

Ingredient (g)	CNO_2_	C0	SA	SMA
Lean muscle tissue	750	750	750	750
Fat muscle tissue	250	250	250	250
Salt	25	25	25	25
Sugar	2	2	2	0
Spice mix ^1^	0.3	0.3	0.3	0.3
Sodium ascorbate	0.5	0	0.5	0.5
Sodium nitrite	0.05	0	0	0
Potassium nitrate	0.08	0	0	0
Polyphenol mix ^2^	0	0	0	0.86
*Coagulase-negative Staphylococcaceae*	0.25	0	0.25	0.25
*Lactic acid Bacteria*	0.125	0	0.125	0.125

^1^ Garlic: black pepper (1:10 *w/w*) ^2^ Grapeseed extract: olive extract: green tea extract (1:1:3 *w/w*) for a total gallic acid equivalent content of 60 g/100 g.

## Data Availability

Not applicable.

## Supplementary Materials

The following supporting information can be downloaded at: https://www.mdpi.com/article/10.3390/ijms232012555/s1.
