# Supplementary material for: Cleaning the Label of Cured Meat; Effect of the Replacement of Nitrates/Nitrites on Nutrients Bioaccessibility, Peptides Formation, and Cellular Toxicity of In Vitro Digested Salami
Source: Int J Mol Sci. 2022 Oct 19;23(20):12555. doi: 10.3390/ijms232012555 (PMC9604274; doi:10.3390/ijms232012555)
Supplement: Supplementary file 1 [file ijms-23-12555-s001.zip › ijms-1968816-supplementary.pdf]

Table S1. Fatty acid methyl esters of undigested salami. Data are means ± SD of three different samples each analyzed in duplicate and are expressed as mol/100mol. Statistical analysis was by the one-way ANOVA (14:0, 18:3 n-3, and 20:1 n-9: $p < 0.05$) with Tukey's post-hoc test (different letters indicate significant differences). C-NO_2: salami with sodium nitrite, potassium nitrate and with nitrate-reducing microbial starter cultures; C-0: salami containing neither nitrate-reducing microbial starter cultures nor additives (nitrite, polyphenols and ascorbate); SA: salami with nitrate-reducing microbial starter cultures and sodium ascorbate; SMA: salami with nitrate-reducing microbial starter cultures, sodium ascorbate and plant extracts.

	**CNO_2**	**C0**	**SA**	**SMA**
14:0	1.6±0.0a	1.5±0.0b	1.6±0.0a	1.6±0.0a
16:0	25.4±0.5a	24.9±0.2a	25.4±0.4a	24.9±0.2a
16:1 n-7	2.5±0.02a	2.5±0.0a	2.4±0.1a	2.4±0.0a
18:0	13.8±0.2a	13.5±0.1a	13.8±0.20a	13.5±0.1a
18:1 n-9	43.4±0.0a	43.5±0.3a	43.9±1.0a	43.5±0.5a
18:2 n-6	11.2±0.5a	11.9±0.3a	10.6±1.5a	11.6±0.2a
18:3 n-3	0.5±0.0b	0.6±0.0a	0.6±0.03a	0.6±0.0a
20:1 n-9	1.0±0.0a	0.9±0.0b	0.9±0.02b	1.0±0.0a
20:4 n-6	0.5±0.1a	0.6±0.1a	0.6±0.09a	0.7±0.0a

Table S2. Fatty acid bioaccessibility of the different salami formulations at each digestion time. Data are means ± SD of in vitro digestion of three independent samples analyzed in duplicate. Fatty acid bioaccessibility is expressed as % and was calculated as fatty acid methyl ester concentration in digested/fatty acid methyl ester concentration in salami before digestion x 100. Statistical analysis was by the one-way ANOVA (18:0 at D60: $p<0.05$) with Tukey's post-hoc test comparing the different experimental salami at each digestion time (different letters indicate significant differences). G120: end of gastric phase; D60: 60 min of duodenal phase; D120: end of duodenal phase. C-NO_2: salami with sodium nitrite, potassium nitrate and with nitrate-reducing microbial starter cultures; C-0: salami containing neither nitrate-reducing microbial starter cultures nor additives (nitrite, polyphenols and ascorbate); SA: salami with nitrate-reducing microbial starter cultures and sodium ascorbate; SMA: salami with nitrate-reducing microbial starter cultures, sodium ascorbate and plant extracts.

	G120				D60				D120			
	CNO_2	C0	SA	SMA	CNO_2	C0	SA	SMA	CNO_2	C0	SA	SMA
14:0	0.5±0.7a	0.3±0.2a	0.4±0.1a	0.7± 0.4a	30.6±4.2a	38.0±6.5a	37.7±1.8a	38.6±2.4a	37.3±0.2a	43.6±6.4a	42.1±3.1a	40.0±0.6a
16:0	0.8±0.4a	0.4±0.2a	1.0±0.1a	0.9±0.5a	29.2±3.0a	39.2±8.3a	32.3±1.1a	40.2±2.2a	37.4±2.7a	45.6±7.9a	43.9±4.2a	37.5±0.0a
16:1 n-7	0.9±0.5a	0.0±0.0a	0.0±0.0a	0.1±0.1a	27.0±4.7a	32.6±5.7a	29.8±3.1a	35.0±2.8a	31.7±0.3a	37.1±5.7a	37.9±4.8a	38.2±2.5a
18:0	0.9±0.6a	0.6±0.1a	1.2±0.1a	1.0±0.3a	28.2±1.2b	40.2±8.9ab	30.8±1.0ab	40.3±1.8a	38.4±8.5a	47.3±10.8a	43.5±4.6a	30.4±2.2a
18:1 n-9	0.6±0.4a	0.2±0.1a	0.8±0.0a	0.6±0.5a	31.0±4.7a	37.2±7.9a	33.0±2.1a	40.0±2.4a	37.5±0.6a	43.1±6.6a	42.4±4.0a	41.8±2.8a
18:2 n-6	0.6±0.5a	0.3±0.1a	0.4±0.1a	0.6±0.4a	36.6±3.8a	39.9±8.7a	42.4±5.8a	44.0±3.5a	45.2±3.0a	47.2±6.3a	54.4±6.2a	48.1±2.1a
18:3 n-3	0.0±0.0a	0.6±1.1a	0.0±0.0a	0.0±0.0a	40.3±14.0a	38.7±19.7a	41.2±8.6a	44.3±6.7a	45.2±4.5a	51.2±6.1a	51.2±4.1a	49.8±1.1a
20:1 n-9	0.0±0.0a	0.0±0.0a	0.0±0.0a	0.0±0.0a	22.1±7.5a	30.1±7.3a	25.0±2.5a	30.4±0.8a	29.3±0.8a	38.1±5.1a	35.6±5.8a	33.4±3.6a
20:4 n-6	0.0± 0.0a	0.0±0.0a	0.0±0.0a	0.0±0.0a	46.3±3.2a	53.0±16.7a	49.5±12.3a	45.0±2.3a	70.3±17.3a	70.0±14.4a	70.3±14.6a	50.8±3.2a
Total	0.6±0.4a	0.3±0.1a	0.8±0.0a	0.7±0.4a	30.6±3.8a	38.3±8.2a	33.3±1.3a	39.9±2.4a	38.3±2.5a	44.7±7.4a	44.1±3.7a	39.8±1.3a

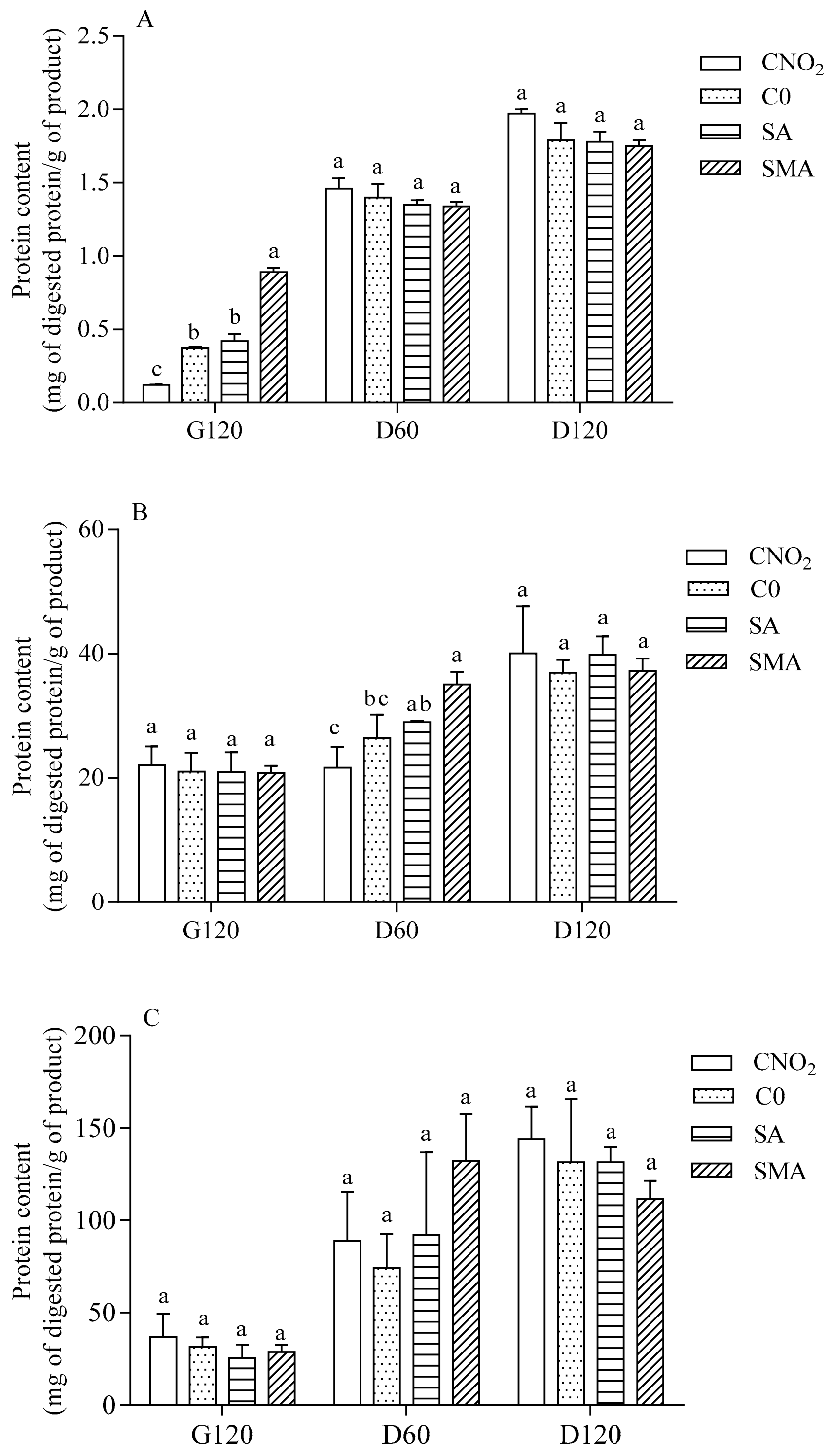

Figure S1. Protein hydrolysys of the different salami formulations at each digestion time. Protein hydrolysis was measured by OPA assay (A), Coomassie assay (B), and by measuring the absorbance at 280 nm (C). Data are means ± SD of *in vitro* digestion of three independent samples analyzed in triplicate. Protein content is expressed as mg of digested protein/g of product. Statistical analysis was by the one-way ANOVA (OPA at G120, and Coomassie at D60 $p < 0.05$) with Tukey's post-hoc test comparing the different formulations at each digestion time (different letters indicate statistical significance). G120: end of gastric phase; D60: 60 min of duodenal phase; D120: end of duodenal phase. $C-NO_2$: salami with sodium nitrite, potassium nitrate and with nitrate-reducing microbial starter cultures; C-0: salami containing neither nitrate-reducing microbial starter cultures nor additives (nitrite, polyphenols and ascorbate); SA: salami with nitrate-reducing microbial starter cultures and sodium ascorbate; SMA: salami with nitrate-reducing microbial starter cultures, sodium ascorbate and plant extracts.

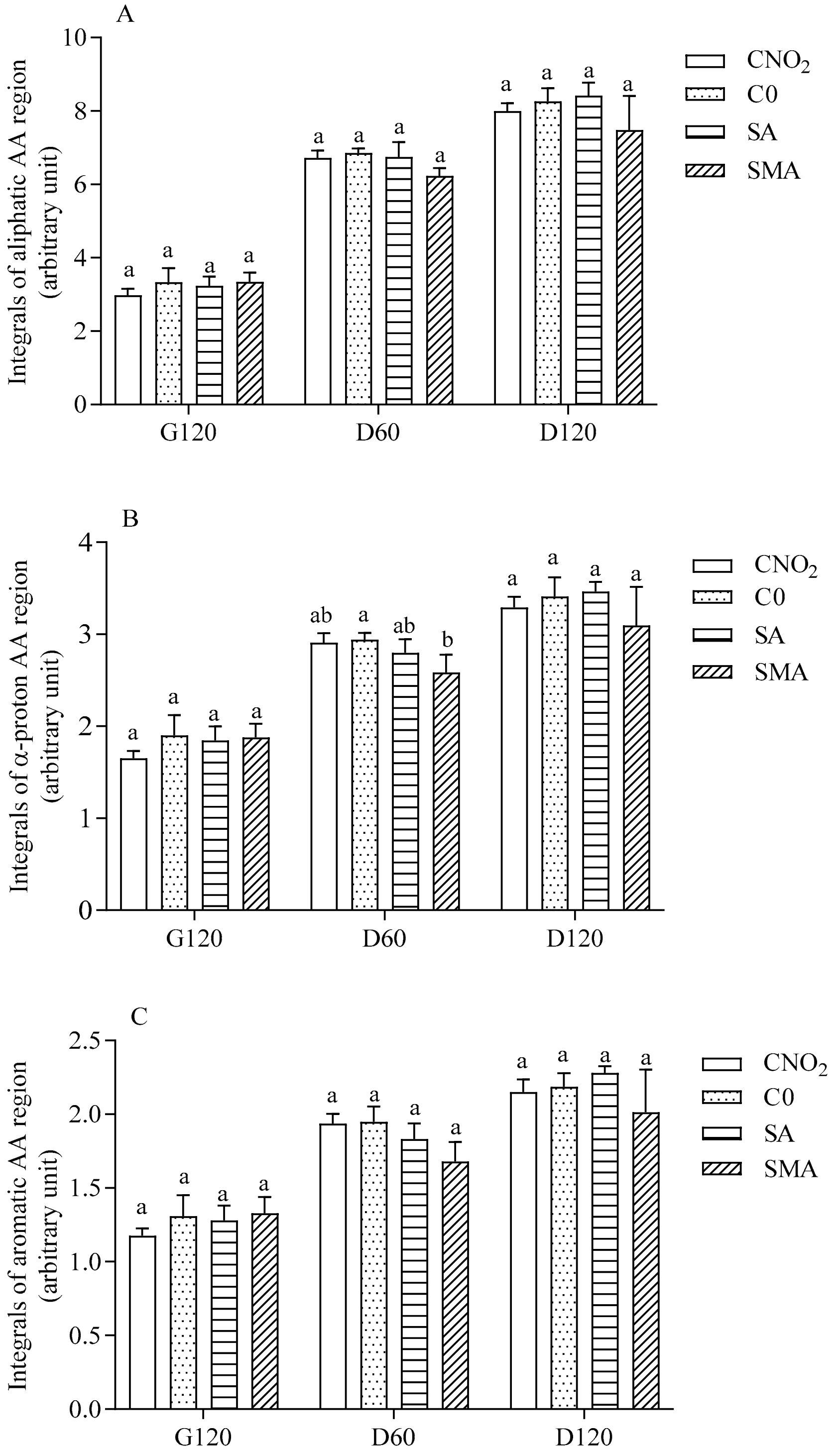

Figure S2. Integral area of aliphatic, α-proton, and aromatic amino acid region of distinct salami at different digestion times. Data are means ± SD of *in vitro* digestion of three independent samples analyzed in duplicate. Integrals of aliphatic (A), α-proton (B), and aromatic (C) amino acid region are expressed as signal area. Statistical analysis was by the one-way ANOVA (B at D60: $p < 0.05$) with Tukey's post-hoc test comparing the different experimental salami in each digestion times (different letters indicate significant differences). G120: end of gastric phase; D60: 60 minutes of duodenal phase; D120: end of duodenal phase; AA: amino acids. C-NO_2: salami with sodium nitrite, potassium nitrate and with nitrate-reducing microbial starter cultures; C-0: salami containing neither nitrate-reducing microbial starter cultures nor additives (nitrite, polyphenols and ascorbate); SA: salami with nitrate-reducing microbial starter cultures and sodium ascorbate; SMA: salami with nitrate-reducing microbial starter cultures, sodium ascorbate and plant extracts.

Table S3. Relative bioactive peptide abundance of salami at different digestion times. Data are means ± SD of not digested or in vitro digestion of three independent samples analyzed in duplicate. Statistical analysis was performed by one-way ANOVA with Tukey's post-hoc test comparing the different formulations before digestion and at each digestion time (different letters indicate significant differences). G120: end of gastric phase; D60: 60 minutes of duodenal phase; D120: end of duodenal phase ND: not digested; DPP-IV: dipeptidyl peptidase 4; ACE: angiotensin-converting enzyme. C-NO_2: salami with sodium nitrite, potassium nitrate and with nitrate-reducing microbial starter cultures; C-0: salami containing neither nitrate-reducing microbial starter cultures nor additives (nitrite, polyphenols and ascorbate); SA: salami with nitrate-reducing microbial starter cultures and sodium ascorbate; SMA: salami with nitrate-reducing microbial starter cultures, sodium ascorbate and plant extracts.

**Peptides sequence**	**Protein source**	**ND**				**ANOVA p value**	**G120**				**ANOVA p value**	**Reported activity (µM IC_{50})**
		**CNO_2**	**C0**	**SA**	**SMA**		**CNO_2**	**C0**	**SA**	**SMA**		
FQPSF	Actin (P68137)	0.0±0.0a	0.0±0.0a	0.0±0.0a	0.0±0.0a	n.s.	2.9±0.5a	2.7±0.2a	3.4±0.1a	3.1±0.1a	n.s.	ACE inhibitor (12.6)
AGDDAPRAVF	Actin (P68137)	0.0±0.0a	0.0±0.0a	0.0±0.0a	0.0±0.0a	n.s.	2.6±0.3a	2.9±0.1a	0.0±0.0b	2.9±0.0a	< 0.05	Bitterness suppressing (n.r.)
AGDDAPR	Actin (P68137)	0.0±0.0a	0.0±0.0a	0.0±0.0a	0.0±0.0a	n.s.	0.0±0.0a	0.0±0.0a	0.0±0.0a	0.0±0.0a	n.s.	Antioxidant (n.r.); ACE (11.9), pancreatic lipase (110.6), and α-amylase (14.7) inhibitor
VAPEHPT	Actin (P68137)	0.0±0.0a	0.0±0.0a	0.0±0.0a	0.0±0.0a	n.s.	0.0±0.0a	0.0±0.0a	0.0±0.0a	0.0±0.0a	n.s.	DPP-IV inhibitor (n.r.)

Peptides sequence	Protein source	D60				ANOVA p value	D120				ANOVA p value	Reported activity (µM IC_{50})
		CNO_2	C0	SA	SMA		CNO_2	C0	SA	SMA		
FQPSF	Actin (P68137)	0.0±0.0a	0.0±0.0a	0.0±0.0a	0.0±0.0a	n.s.	0.0±0.0a	0.0±0.0a	0.0±0.0a	0.0±0.0a	n.s.	ACE inhibitor (12.6)
AGDDAPRAVF	Actin (P68137)	0.0±0.0a	0.0±0.0a	0.0±0.0a	0.0±0.0a	n.s.	0.0±0.0a	0.0±0.0a	0.0±0.0a	0.0±0.0a	n.s.	Bitterness suppressing (n.r.)
AGDDAPR	Actin (P68137)	2.7±0.0c	3.8±0.0b	4.3±0.3a	3.7±0.0b	< 0.05	3.1±0.2a	2.9±0.3a	3.4±0.1a	2.8±0.1a	n.s.	Antioxidant (n.r.); ACE (11.9), pancreatic lipase (110.6), and α-amylase (14.7) inhibitor
VAPEHPT	Actin (P68137)	3.0±0.3a	3.7±0.5a	3.6±0.2a	3.1±0.0a	n.s.	3.1±0.3a	2.7±0.0b	3.2±0.1a	2.7±0.0b	< 0.05	DPP-IV inhibitor (n.r.)

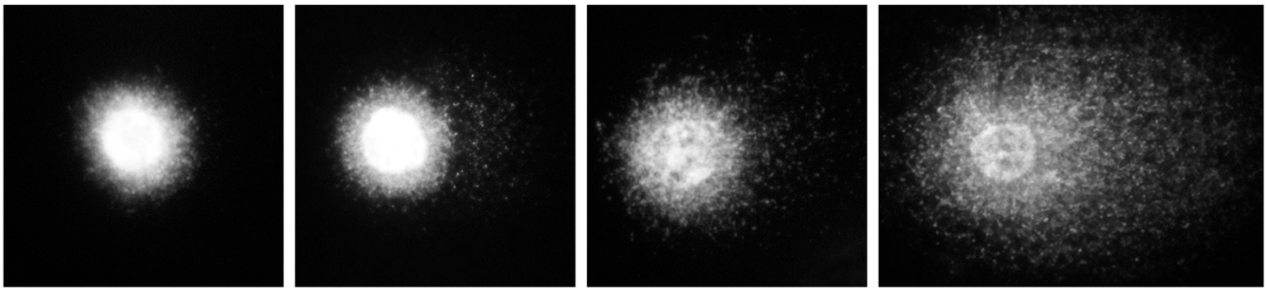

Figure S3. Microscopy images of HT-29 cells were acquired using a Leica DMLS fluorescence microscope (excitation filter BP 515–560 nm, barrier filter LP 580 nm) and an automatic image analysis system (Comet Assay IV—Perceptive Instruments Ltd., Bury St Edmunds, UK). Images provide different levels of DNA damage in HT-29 cancer cells after Comet assay. From left to right we reported an increasing of DNA damage.
